# Morphogenetic and Histogenetic Roles of the Temporal-Spatial Organization of Cell Proliferation in the Vertebrate Corticogenesis as Revealed by Inter-specific Analyses of the Optic Tectum Cortex Development

**DOI:** 10.3389/fncel.2016.00067

**Published:** 2016-03-17

**Authors:** Melina Rapacioli, Verónica Palma, Vladimir Flores

**Affiliations:** ^1^Interdisciplinary Group in Theoretical Biology, Department of Biostructural Sciences, Favaloro UniversityBuenos Aires, Argentina; ^2^Laboratory of Stem Cell and Developmental Biology, Faculty of Science, University of ChileSantiago, Chile

**Keywords:** cell proliferation, corticogenesis, morphogenesis, histogenesis, patterning

## Abstract

The central nervous system areas displaying the highest structural and functional complexity correspond to the so called cortices, i.e., concentric alternating neuronal and fibrous layers. Corticogenesis, i.e., the development of the cortical organization, depends on the temporal-spatial organization of several developmental events: (a) the duration of the proliferative phase of the neuroepithelium, (b) the relative duration of symmetric (expansive) versus asymmetric (neuronogenic) sub phases, (c) the spatial organization of each kind of cell division, (e) the time of determination and cell cycle exit and (f) the time of onset of the post-mitotic neuronal migration and (g) the time of onset of the neuronal structural and functional differentiation. The first five events depend on molecular mechanisms that perform a fine tuning of the proliferative activity. Changes in any of them significantly influence the cortical size or volume (tangential expansion and radial thickness), morphology, architecture and also impact on neuritogenesis and synaptogenesis affecting the cortical wiring. This paper integrates information, obtained in several species, on the developmental roles of cell proliferation in the development of the optic tectum (OT) cortex, a multilayered associative area of the dorsal (alar) midbrain. The present review (1) compiles relevant information on the temporal and spatial organization of cell proliferation in different species (fish, amphibians, birds, and mammals), (2) revises the main molecular events involved in the isthmic organizer (IsO) determination and localization, (3) describes how the patterning installed by IsO is translated into spatially organized neural stem cell proliferation (i.e., by means of growth factors, receptors, transcription factors, signaling pathways, etc.) and (4) describes the morpho- and histogenetic effect of a spatially organized cell proliferation in the above mentioned species. A brief section on the OT evolution is also included. This section considers how the differential operation of cell proliferation could explain differences among species.

## Basic Organization of the Developing Central Nervous System

The developing vertebrate central nervous system (CNS) is composed of distinct regions aligned along the cephalic-caudal (Cph-Cd) axis: Forebrain, Midbrain, Hindbrain, and Spinal Cord. Early in development, each prospective region is composed of two main populations of neural stem cells (NScs) characteristically located along the dorsal-ventral (Dor-Ven) axis: the alar (dorsal) and the basal (ventral) plates that originate associative and efferent neuronal populations, respectively. Each encephalic region (fore-, mid-, and hindbrain) originate, amongst other structures, multilaminated concentric organizations of alternating neuronal and fibrous layers, generically named as “cortices” or “cortex”: the cerebral cortex or *cortex cerebri* (derived from the dorsal telencephalon of the prosencephalic alar plate), the tectal cortex or *cortex tecti mesencephali* (derived from the mesencephalic alar plate) and the cerebelar cortex or *cortex cerebeli* (derived from the rhombic lips of the metenchepalic region of the post-encephalon). Cortices exhibit the highest structural and functional organization of the CNS. They receive afferent information from multiple origins, process and integrate the afferent information and elaborate complex responses.

## Structure and Function of the Midbrain Tectum

**FIGURE 1:**
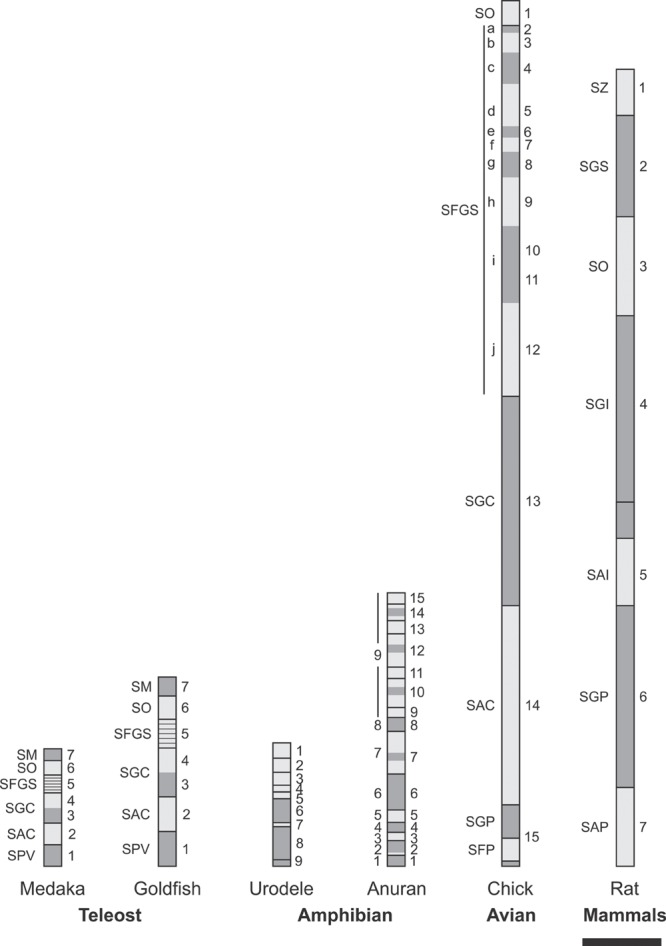
**Homologies in vertebrate OT radial organization.** Vertebrates OT show a basic multilayered organization consisting of alternating neuronal (dark gray) and fibrous (light gray) laminae. It is assumed that this basic pattern results from successive phases of mitotic activity and post-mitotic neuronal migration. The striking simplicity of urodele is interpreted as a paedomorphic suppression of the post-mitotic neuronal migration. SM, Stratum marginale; SO, stratum opticum; SFGS, stratum fibrosum et griseum superficiale; SGC, stratum griseum centrale; SAC, stratum album centrale; SPV, Stratum periventriculare; SGP, stratum griseum periventriculare; SFP, stratum fibrosum periventriculare; SZ, stratum zonale; SGI, stratum griseum intermedium; SAI, stratum album intermedium; SGP, stratum griseum profundum; SAP, stratum album profundum. Scale bar: 100 μm. Redrawn and modified from Meek and Schellart (1978), Altman and Bayer (1981), Raymond and Easter (1983), Butler and Hodos (2005), and Luksch (2009).

The tectum mesencephali (midbrain tectum) is a center for processing of sensory (auditory, somatosensory, visual) inputs. In most species the visual component is the most important and for that reason it is commonly named as optic tectum (OT). It is composed of several alternating neuronal and fibrous layers whose names refer to their positions along the radial axis and their most relevant morphological features. The OT and the Torus semicircularis are homologs of the mammal’s superior and inferior colliculus (SC and IC) respectively. **Figure 1** shows the similarities in the organization of the OT in several species.

The OT is composed of a large variety of neuronal types located at different layers but, as a common rule, they belong to one of two basic categories: **(a)** Macroneurons are born early and differentiate into large multipolar efferent neurons with long axons that project to extrinsic OT targets and form projection pathways and **(b)** Microneurons are born later and differentiate into small associative interneurons with short axons that ramify within the OT and form intrinsic local circuits (Potter, 1969; LaVail and Cowan, 1971a; Altman and Bayer, 1981; Roth et al., 1993).

Time and position at which each neuronal cohort appears during the OT development are crucial factors in determining the type of adult neurons they will originate and the positions of these neurons over the tangential plane and along the radial axis of the adult OT cortex.

## The Temporal-Spatial Organization of the Proliferative Activity. Its Role in Morpho- and Histogenesis: From Teleosts to Mammals

It is considered that the close relationships between “time and place of birthday” and developmental fate and position arises from the fact that the proliferative activity is, by itself, temporally and spatially organized and that this organization is instrumental in modeling the OT cortex and its lamination. In fact, it is generally accepted that differential or asymmetric growth is a relevant process in the production of shape changes in a developing system. It is known that the differential planar expansion of the neuroepithelium, combined with restrictions to expansion at specific zones, contribute to model the neural tube morphology (Concha and Adams, 1998; Ciruna et al., 2006; Kriegstein et al., 2006).

### Teleosts Fishes

It is commonly considered that the simpler the CNS organization the clearer is the relationship between the spatiotemporal organization of cell proliferation and morpho-histogenesis (Northcutt, 1983; Vanegas and Ito, 1983). Due to their simplicity and smallness, the teleosts OT shows well-defined, spatially organized, zones containing proliferating NScs, early post-mitotic neurons and differentiating neurons. This feature makes the developing teleosts OT widely used models of the morphogenetic role of cell proliferation (Raymond and Easter, 1983; Fernald, 1990; Vecino, 1998; Hu and Easter, 1999; Li et al., 2000). Apart from some interspecific differences, the teleosts OT share a common basic spatiotemporal pattern of NScs proliferation. As an illustrative example of that pattern, **Table 1** offers a sequence of developmental events in the Medaka OT (Nguyen et al., 1999).

**Table 1:** Sequence of developmental stages in the medaka embryo OT (Nguyen et al., 1999).

Developmental stage	Principal events
DS22	Tectal plate (OT primordium) formation
DS22–DS26	The tectal plate enlarges uniformly. The entire NScs population proliferates at a similar rate
DS26–DS27	A superficial post-mitotic zone (sz) appears at the cephalic and lateral zone of the tectal plate. The remaining structure retains proliferative activity
DS27–DS28 onward	Marginal proliferative zone (Mpz) formation. Proliferation retreats to a crescent-shaped zone along the OT periphery (medial, caudal, and lateral margins). This zone plays a crucial neuronogenic function
DS29–DS30	Beginning of lamination. An external fibrous layer becomes apparent at the cephalic-lateral region. The Mpz is better defined
DS31	Beginning of neuronal radial migration. Radially, migrating neurons invade the cephalic zone of the external (fibrous) layer. The radial migration and differentiation progress along a cephalic-lateral→caudal-medial spatial gradient. The most differentiated area is the cephalic-lateral pole
DS39 (hatching)	The layers of the adult OT (central zone (cz), superficial zone (sz) and periventricular gray zone (pgz)) are distinguishable
Post-hatching	NS cells persist at the Mpz allowing a postnatal period of neuronogenesis. Proliferation gradually declines

**FIGURE 2:**
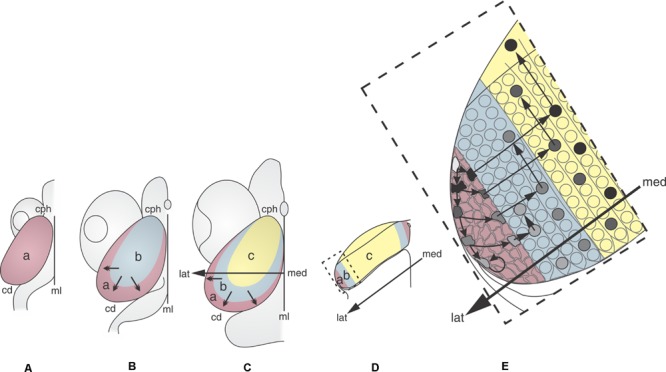
**2D representations of the dorsal aspect of the medaka embryo OT at different developmental stages (DS).** (A: DS22–DS26; B: DS30; C: DS32–39). The sequence illustrates the distribution of the NS cells (purple) during the OT grow and differentiation. Neuronal cohorts born at different DS acquire different positions. **(A)** DS22–26. The entire OT proliferates at the same rate. **(B)** DS30. Post-mitotic neurons that were born between DS22–26 and DS30 occupy the central region (light blue). The remaining NS cells are located at the peripheral zone. **(C)** DS39. Post-mitotic neurons that were born between DS32 and DS39 occupy a crescent shaped area (yellow), NS cells are located peripherally. **(D)** Traverse section through the med-lat axis shown in **(C)**. **(E)** Higher magnification of the box shown in **(D)**. Cells with different labeling intensity (black to light gray) can be seen after a single pulse of thymidine. Black cells correspond to those that exit the cell cycle and the proliferation zone immediately after labeling. Cells with decreasing tones of gray (gray tones) represents cells that do not exit the cell cycle and remains in the proliferation zone for 2–4 additional cell cycles. The post-mitotic neurons that exit the proliferation zone during the early stages (black circles) occupy the central zone and start differentiation and radial migration. Subsequently, new columns of post-mitotic neurons are added from the peripheral zone. The newly born neurons, after entering the central zone, begin their radial migration and differentiation. a: zone of proliferative NS cells (purple); b: zone of newly-born neurons (blue); c: zone of differentiated neurons (yellow). Med→lat arrows: medial-lateral axis. Small arrows: direction of the OT tangential expansion. Redrawn and modified from Nguyen et al. (1999).

**Figure 2** shows that the OT neuronogenesis, tangential expansion and histogenesis depend on how NScs, post-mitotic cells, migrating neurons, and differentiating neurons are spatially organized in the OT primordium.

During the early phase (DS22 to DS26–27), the whole tectal plate (**Figure 2Aa**) is composed of NScs that proliferate uniformly. The post-mitotic neurons generated during this period occupy the central zone (**Figure 2Bb**) and the NScs are gradually displaced to the peripheral zone. Thus, a marginal proliferative zone (Mpz; zone “a” in **Figure 2B**), occupied exclusively by NScs, forms a ring around the central zone “b.” From this time onward cell proliferation becomes restricted to the Mpz. This spatial organization results in a centrifugal tangential expansion of the OT primordium. From DS27–28 onward -throughout the transitional phase DS29–31- the earliest born neurons begin their radial migration. This process follows a cephalic-lateral→caudal-medial axis. From DS30–32 onward cells with different “developmental status” can be seen spatially organized over the OT tangential plane (zones “c,” “b,” and “a” in **Figure 2C**): (1) a central zone “**c**” occupied by differentiating and migrating neurons; (2) a zone “**b**” occupied by cells exiting the cell cycle (newly born post-mitotic neurons); and (3) a zone “**a**” or Mpz populated by two kinds of NScs (rapidly and slowly proliferating cells).

During the last phase (planar growth) the OT expands tangentially thanks to the continuing activity of the Mpz. This phase continues post-hatching and involves NScs proliferation, neuronal migration, and differentiation. After hatching, there is gradual decrease in the number of rows of proliferating NScs at the Mpz (in Medaka: 10–12 rows at DS30; 5–6 at hatching and to 1–3 in adult). Post-hatching cell proliferation in the OT has been documented in several teleosts [in goldfish (Raymond and Easter, 1983), trout (Mansour-Robaey and Pinganaud, 1990), zebrafish (Wullimann and Knipp, 2000; Loeb-Hennard et al., 2005; Feijóo et al., 2011; Jung et al., 2012)].

Studies of cell tracing, with BrdU labeling, show that the OT tangential growth results from the addition of successive neuronal cohorts generated at the Mpz (**Figure 2E**). This figure illustrates that, after performing their last mitosis at the Mpz (zone “**a**”), successive cohorts of post-mitotic cells are added, as concentric columns (zone “**b**”), around the central differentiated zone “**c.**” Once these neurons access (enter) the central zone, they begin their radial migration and differentiation.

These studies demonstrate that, during the OT cortex lamination, the BrdU+ differentiating neurons are organized as radial columns indicating the absence of a significant tangential dispersion during their radial migrations. This feature is considered a basic evolutionary characteristic common to all vertebrates including higher mammals. These patterns of developmental events basically coincides with those reported in diverse teleosts using different methods of mitotic cells labeling: rainbow trout (Mansour-Robaey and Pinganaud, 1990); post-embryonic zebrafish and medaka (Nguyen et al., 1999, 2001; de Oliveira-Carlos et al., 2013); adult Apteronotus (Zupanc and Horschke, 1995); adult Gasterosteus aculeatus (Ekström et al., 2001); brown trout (Candal et al., 2005).

### Amphibians

The amphibians OT underwent striking evolutionary changes with respect the teleosts OT. As an example, the anurans OT are significantly thicker and show more complex organization and stratification than the teleosts OT (**Figure 1**; Schmidt and Roth, 1993). There are also significant structural differences between the orders anurans and urodeles within the class amphibians. These differences can be illustrated by comparing the OT organization in *Pleurodeles waltl* and *Rana temporaria*. *Pleurodeles* OT possesses a simple laminar structure (a periventricular neuronal layer and a superficial fibrous layer) while the *Rana* OT cortex shows a multi-stratified organization (nine, alternating, neuronal, and fibrous layers; Potter, 1969; **Figure 1**). This difference has been interpreted as a paedomorphic simplification resulting from a suppression of the radial neuronal migration in *Pleurodeles* (Roth et al., 1993). It has been proposed that several specific structural differences between the OT of these species could be due to quantitative differences in proliferation (Schlosser, 2008). It is also likely that many histological differences between both species derive from differences in the spatial organization of cell proliferation during the OT morpho-histogenesis. For instance, *P. waltl* have a lower proliferation rate in the lateral proliferative zone, and this lead to a reduction in the lateral OT region with respect to *R. temporaria*.

Despite the above-mentioned differences in OT organization and complexity, there are some basic similarities in the pattern of proliferation in the developing OT in salamander (*P. waltl*) and frogs (*R. temporaria*; Schmidt and Roth, 1993). Besides, the temporal-spatial organization of neuronogenesis in amphibians, more or less, follows the pattern observed in teleosts fishes although with significant differences in complexity and morphogenetic results. During the early development of the OT primordium in both *P. waltl* and *R. temporaria*, the proliferative activity is uniformly distributed over the whole ependymal layer. Later, as development progresses, the NScs organize their proliferative activity along the cph-lat→cd-med axis. Finally, proliferation remains confined to a reduced band along the dorsal-caudal-medial OT margins in both species.

**FIGURE 3:**
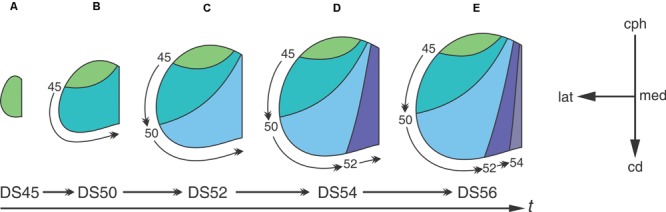
**The temporal sequence of diagrams represents the progression through developmental stages.** The progression from **(A–E)** shows that the tangential growth of the OT wall depends on the temporal-spatial organization of NScs proliferation. Neurons subpopulations born at different DS -different time intervals- occupy different regions of the OT tangential plane. Curve lines represent boundaries between regions of the tangential plane occupied by cohorts of neurons born at different DS. Arabic numbers indicate the DS at which 3H-TdR was administered. The rostral-most area (green) corresponds to a region occupied by neurons that were born before the first labeling experiment. The others four colored areas represent regions of the OT tangential plane formed between two successive labeling experiments. The small curved arrows represent the extension of the tangential plane formed after each labeling (the time of label administration is indicated at the origin of each arrow). The orthogonal reference system cph→cd/med→lat represents the tangential plane. Redrawn and modified from Straznicky and Gaze (1972).

Analyses of neuronogenesis in another anuran, *Xenopus laevis*, show that the OT neurons are born in a topographical order from the cephalic-ventral to the caudal-medial OT regions (Straznicky and Gaze, 1972). Autoradiographic analyses with methyl-tritiated thymidine (3H-TdR), administered at different DS, allowed the construction of a “neuronogenic map” that correlates the time of neuronal birthday with their position along the cph→cd axis. **Figure 3** shows that: (1) the neurons located at the rostro-ventral pole of the adult OT undergo their last mitosis between DS35–45-; (2) neurons comprising the OT ventro-lateral border exit the cell cycle after DS45 and (3) those located at the caudo-medial region are born between DS50–55. The final part of the OT to form is the caudal-medial part of the dorsal roof and this takes place by stage 58. After DS58, no neuron is added to the OT cortex. It has been reported that NScs persist in the adult torus semicircularis in *Rana esculenta* (Raucci et al., 2006) and *Rana catesbeiana* (Simmons et al., 2008).

**Figure 3** graphically illustrates the relationship between the spatial organization of cell proliferation during development and the neuronal distribution in the adult OT. The “neuronogenic map” shows that neurons localized at different positions along the cph-cd axis of the adult OT are born during successive DSs. This temporal-spatial organization is because the addition of cohorts of cells (stripes of wall) at the caudal-medial zone displaces the pre-existing tissue laterally and cephalically.

### Birds

The development of the bird OT has been exhaustively studied in several species (pigeon, zebra finch, owls, chicks, ducks, etc.; LaVail and Cowan, 1971a,b; Heaton and Munson, 1978; Bagnoli et al., 1987; Herrmann and Bischof, 1993; Manns and Güntürkün, 1997; Mey et al., 1998; Mey and Thanos, 2000; Heidmann and Luksch, 2001; Luksch, 2003; Nieder et al., 2003). During early stages the proliferative activity is uniformly distributed in the midbrain primordium. But from embryonic day 2 (ED2) onward, with the appearance of the isthmic organizer (IsO), an asymmetric distribution in mitotic density is installed along the cph→cd axis. Actually, it was known for many years that the density of mitotic cells is consistently higher in the caudal half (Fujita, 1962, 1964, 2003; Cowan et al., 1968; Wilson, 1971, 1973).

**FIGURE 4:**
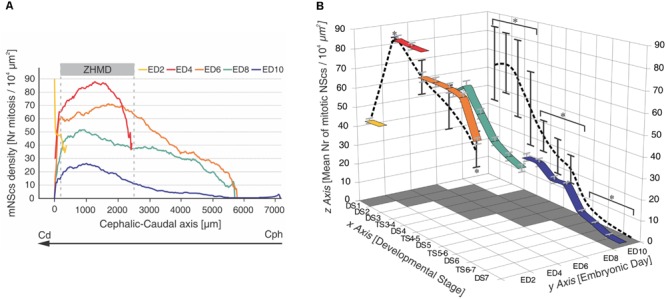
**Temporal-spatial organization of NSc proliferation in the chick embryo OT. (A)** Changes in NScs proliferation as a function of time and space in the developing chick OT. Each curve represents a smoothened profile of mitotic NScs (mNScs) density along the cph-cd axis and obtained at different embryonic days (ED). A zone of high mitotic density (ZHMD) localizes, from ED4 onward, at a constant position from the caudal midline (between 200 and 2500 μm). The maximal mitotic density occurs at ED4. Between ED2 and ED6, the ZHMD is mainly occupied by NScs that divides symmetrically (expansive phase). From ED6 onward, the ZHMD is occupied by NScs that start asymmetric divisions. Cephalic–caudal (cph-cd) vector: developmental gradient (DG) axis. Modified from Rapacioli et al. (2012b). **(B)** Tridimensional mesh graph of means values of mitotic NSc density corresponding to the different developmental stages (DSs) and transitional stages (TSs) found along the developmental gradient axis at different embryonic days (EDs). The “x-y” plane, i.e., DS, ED plane, represents a time-space window because the series of DSs corresponds to sequences of segments of the developmental gradient axis. The values of the “z” axis within the “x,y,z” cube correspond to the mean ± standard deviation estimated for each DS at each ED [mean number of mitotic NScs/10^4^ μm^2^]. The colored ribbons represent variations in mitotic density (“z” axis) as a function of the DSs (“x” axis) recorded at different EDs (“y” axis). The values of the “z” axis represented on both the “xz” and the “yz” planes correspond to the mean ± standard deviation of the means values indicated within the cube. ^∗^Statistically significant differences were found within both planes. Three clusters of values (DS1 – TS3–4), (DS4–DS5), (TS5–6 – DS7) with significant differences amongst them were found on the x–z plane (*p* < 0.001). Three clusters of values (ED2, ED6, ED8), (ED4), (ED10) with significant differences amongst them were found on the y–z plane (*p* < 0.001). Modified from Rapacioli et al. (2011).

**FIGURE 5:**
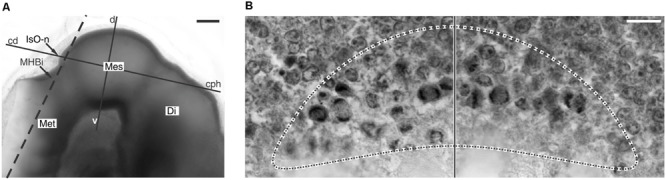
**Cell proliferation spatial organization and the isthmic organizer node. (A)** Whole-mount preparation of the chick embryo encephalon. The dashed line shows the orientation of a plane of section tangential to the IsO node. **(B)** Histological section with the orientation shown by the dashed line in A. It is a tangential section through the IsO node. The dotted line encircles the medial ZHMD around the cephalic border of the IsO node. Vertical line: sagittal plane. Di, Diencephalon; Mes, Mesencephalon; Met, metencephalon; cph-cd and d-v lines, orientations of the cephalic-caudal and dorsal-ventral axes respectively. Modified from Rapacioli et al. (2012b). Scale bars: **(A)** 100 μm; **(B)** 10 μm.

**FIGURE 6:**
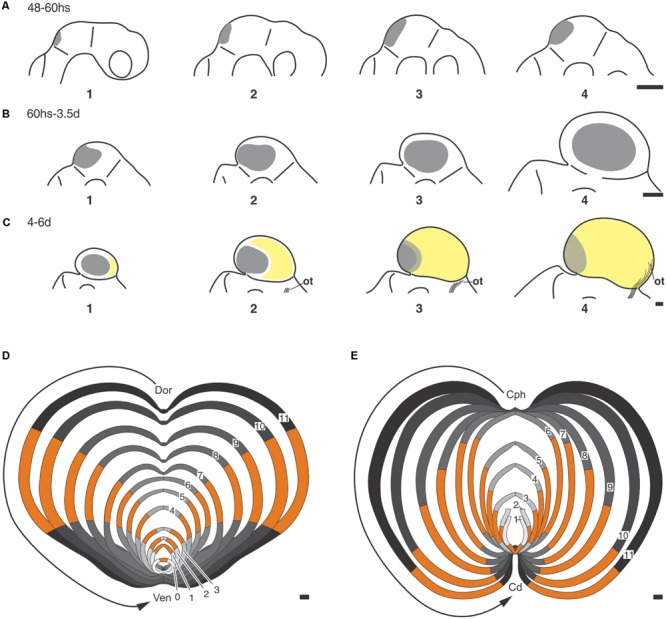
**(A–C)** Positional changes underwent by the ZHMD during the OT morphogenesis (Chick embryo OT). **(A)** (1–4) A medial ZHMD (gray area) appears at the IsO zone. The ZHMD elongates along the dorsal midline and form a dorsal convexity. **(B)** (1–4) The medial ZHMD is replaced by two lateral ZHMD that “move” toward the central region of each hemitectum. **(C)** (1–4) Relative caudal displacement of the lateral ZHMD due to the appearance of a large “neuronogenic zone” (yellow area) at the cephalic end, i.e., the zone where the optic tract (ot) enter the OT cortex. From ED5–6 onward, both symmetric (expansive) and asymmetric (neuronogenic) proliferation occurs at the ZHMD. **(A–C)** Were drawn with different magnifications. Scale bars = 20 μm. **(D,E)** Schematic representation of the step-by-step morphogenetic model based on the temporal–spatial organization of NScs proliferation (Chick embrio OT). The sequence of images (1–11) shown in **(D,E)** illustrate the positional changes underwent by the ZHMD (orange) and their associated morphological changes. **(D)** Composition of dorsal-ventral sections. **(E)** Composition of cephalic-caudal sections. These compositions consist of graphic representations of the changes described in **Table 2**. Dor→Ven vector: dorsal-ventral axis; Cph→Cd vector: cephalic-caudal axis. All images (1–11) were drawn with the same magnification. Scales: 100 μm. Modified from Rapacioli et al. (2012b).

A recent characterization of DS shows that the OT development is highly dynamic (Rapacioli et al., 2011): (a) there is an asymmetric progression of DS due to the fact that they progress as a function of time and also propagate along a cph-ven-lat→cd-dor-med developmental gradient axis; (b) for that reason, several spatially organized, DSs are simultaneously present at any ED or any Hamburger and Hamilton (1951; HH) embryonic stage; (c) this organization is because several cell behaviors (NScs proliferation, neuronal determination, post-mitotic migration, and differentiation) are temporally and spatially organized and progress according the above mentioned developmental gradient (Rapacioli et al., 2012b; **Figures 4–6**).

It was shown that in the chick OT the NScs proliferation is organized around the IsO. By ED2 a medially located zone of high mitotic density (mZHMD) appears around the IsO node (**Figure 5**).

From E2 onward the mZHMD undergoes a series of relative positional changes (**Figure 6**) and each of them correlates with a simple but significant morphogenetic change. These correlations are temporally organized into a proliferation-based step-by-step model of OT morphogenesis summarized in **Table 2** (Rapacioli et al., 2012b).

**Table 2:** Sequence of positional changes underwent by ZHMDs and their corresponding morphogenetic effects (Rapacioli et al., 2012b).

Step	Principal events
Step 0	Mid-hind brain (M-H) isthmus and IsO formation. This process occurs before E2 (10–13 somites to 19–21 somites). The IsO cells organize around the cephalic-lateral border of the IsO node (Alvarez Otero et al., 1993; Alexandre and Wassef, 2003; Louvi et al., 2003)
Step 1	Establishment of a mZHMD at the IsO zone. By E2, the zone of the IsO node transforms into the mZHMD (**Figures 5** and **6A1,D1,E1**)
Step 2	Dorsal midline elongation (**Figure 6D1–4**). Duplication of the mZHMD and constitution of two bilateral bZHMD (**Figures 6A4–B3,D1–3,E1–3**)
Step 3	The bZHMDs “move” cephalically and ventrally toward the central region of each hemitectum (**Figures 6D3–6,E3–6**)
Step 4	Lateral expansion, Intertectal fissure formation and separation of the left and right OT hemispheres (**Figures 6C1–4,D6–11**)
Step 5	Relative “displacement” of the bZHMDs toward the caudal region (**Figures 6C1–4,D6–11**)

The most remarkable change the OT undergoes during the proliferative phase is the intertectal sulcus formation and the segregation of the OT into two bilateral halves: the left and the right tectal hemispheres. This morphogenetic change was attributed to space-dependent differences in cell proliferation: a reduction in cell proliferation along the dorsal midline associated to a concomitant increase in proliferation in both lateral regions (Rapacioli et al., 2012a). This hypothesis was tested by the electroporation of the OT dorsal region with either Sonic hedgehog (Shh) or its main downstream effector, the transcription factor (TF) Gli1. Indeed, this procedure increased notably NScs proliferation along the dorsal midline and abolished the intertectal sulcus formation. No proliferative activity was detected in the chick OT after hatching (Alvarez-Buylla and Lois, 1995; Mezey et al., 2012).

### Mammals

The mammalian midbrain tectum is composed of the SC (homologous of the OT of fishes, amphibians, and birds) and the IC (homologous of the torus semicircularis). The mammalian SC is a multilaminated structure composed of (**a**) superficial layers (stratum zonale, stratum opticum and stratum griseum intermedium), (**b**) intermediate layers (stratum album intermedium) and (**c**) deep layers (stratum griseum profundum and stratum album profundum; Altman and Bayer, 1981; Wallace, 1988; Qu et al., 2006). These zones differ in structure, connections and physiology (Gordon, 1973; Antonetty and Webster, 1975; Dräger and Hubel, 1975; May, 2006).

Autoradiographic labeling with 3H-TdR performed at different stages in the albino *Rattus norvegicus* SC show that neurons born at different developmental stages give origin to different neuronal types and populate different cortical laminae in the adult stage (Mustari et al., 1979). **Table 3** summarizes data about the relationships between (**a**) neuronal birthdays and (**b**) neuronal type and position. Studies of cell tracing performed in rat embryos indicate that: (**a**) the entire populations of OT neurons are generated during a 5 days interval (ED12 to ED17), (**b**) neurons are born according to a cephalic-lateral-ventral→caudal-medial-dorsal spatial gradient and (**c**) neurons located at the deeper layers (SGP) are generated earlier that those located superficially (SGFS).

**Table 3:** Relationship between time of origin, neuronal type, and radial position in the rat embryo SC.

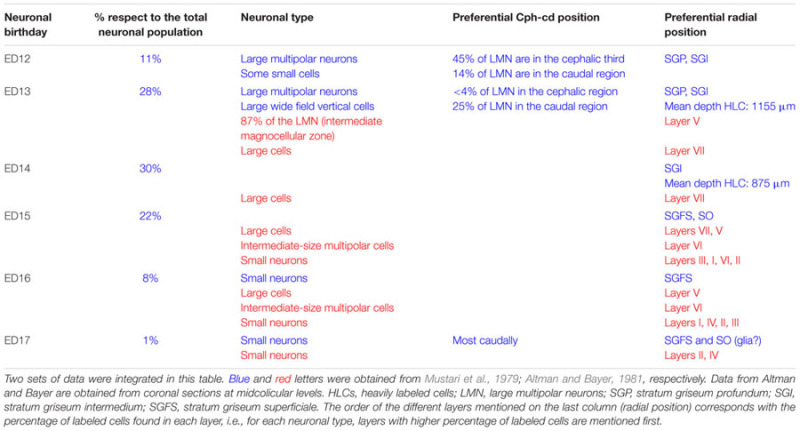

**FIGURE 7:**
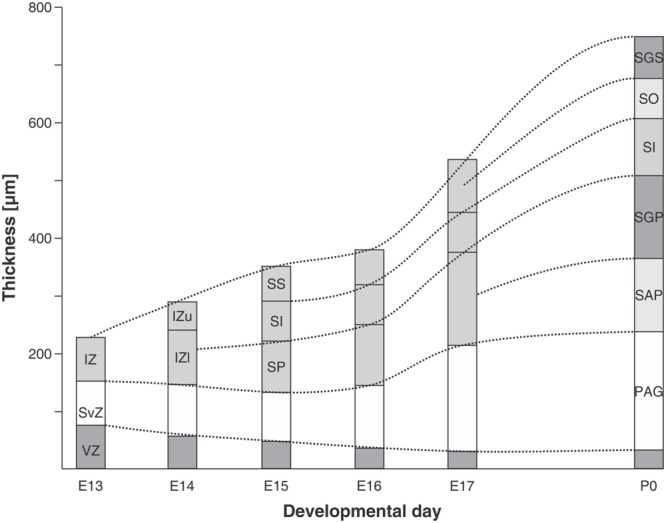
**Chronology of stratification in the mouse embryo SC.** Initially the SC neuroepithelium is a generation zone composed of ventricular zone (VZ) and subventricular zone (SvZ). At E13 the intermediate zone (IZ) appears and at successive ages becomes subdivided into different layers. At E14 the IZ is divided into upper (IZu) and lower (IZl) zones. During the following days the stratification continues and by P0 the definitive layers (denoted by their adult terminology) can be identified. SS, stratum superficiale; SI, stratum intermedium; SP, stratum profundum; SGS, stratum griseum superficiale; SO, stratum opticum; SI, stratum intermedium; SGP, stratum griseum profundum; SAP, stratum album profundum; PAG, periaqueductal gray. Modified from Edwards et al. (1986).

In the rat embryo SC, neuronogenesis begins on ED12 and the first neuronal cohorts corresponds to the large multipolar neurons that later reside in layers V and IV. The temporal sequence of generation of neuronal cohorts correlates with their position (stratification) along the radial axis. The stratification progresses according to a spatial developmental axis and is similar to those described in the OT of the chick and other non-mammalian vertebrate species. A precise correlation between time of origin of neuronal subpopulations and stratification was also reported in the mouse (Edwards et al., 1986). In fact, the vast majority of neurons are born between ED11–ED14 and each lamina has a preferential period of neuronogenesis: SAP, E11; SGP, ED11–ED12; SAI, ED11–ED12; SGI, ED12–ED13; SO, ED12; SGS, ED13. Relatively few neurons are born after ED14. A well-defined stratification of these subpopulations begins at ED13 and follows a clear temporal order (**Figure 7**).

In the gold hamster (*Mesocricetus auratus*) embryo, the neurons of the SC are born between E10.5 and E12. Similar to the pattern observed in the rat, the neurons of the deepest laminae of the SC are born during the early stages and those composing the intermediate and superficial layers are born later (Crossland and Uchwat, 1982). Besides, the large neurons are born earlier and during the later stages there is a shift toward smaller neurons (Crossland, 1987). The neuronogenesis is organized according to a clear cephalic→caudal gradient and a weak lateral→medial gradient is also observed.

Autoradiographic labeling with 3H-TdR performed in rhesus monkey (*Macaca mulatta*) show similar patterns but the above mentioned neuronogenic gradients are not so conspicuous as in other mammalian species (Cooper and Rakic, 1981). The neurons of the SC are born between E30 and E56, with a peak of cellular proliferation between E38 and E43. Although, a rostral→caudal gradient is not apparent at the onset of neuronogenesis, a weak gradient emerges by the end of the process; the neurons that are born from E43 onward preferentially occupy the caudal regions. There is also a weak deep→superficial gradient: those neurons that are born during the first phase preferentially occupy the deep layers while those that are born from E48 onward preferentially populate the more superficial layers.

## Molecular Regulation of the OT Development. OT Patterning and Spatial Organization of Cell Proliferation

The elaboration of the OT multilayered organization, with each layer composed of different combinations of several neuronal types that are born at different developmental stages, requires the generation of the appropriate number of neurons for each neuronal type at defined times and positions. This temporally and spatially organized neuronogenic process depends on two different sets of regulatory mechanisms: (**a**) an initial patterning produces a spatially organized distribution of distinct NScs and neuroprogenitor cells populations with committed regional identity and (**b**) the establishment of different proliferation dynamics with space-dependent specificities.

### Patterning

#### Induction, Maintaining, and Positioning of the Isthmic Organizer

The IsO located at the midbrain-hindbrain (M-H) boundary, is a highly conserved secondary organizing center typical of vertebrates. The IsO installs the polarity of the OT primordium, organizes the OT morpho-histogenesis and controls the OT retinotopic map (Joyner, 1996; Joyner et al., 2000; Echevarría et al., 2003; Mason, 2007; Ishikawa et al., 2008; Agoston et al., 2012).

Several steps are considered to occur between the time when the position of the future IsO is determined and the time when a combinatorial operation of several cell behaviors translates the positional information provided by IsO into a structural, morpho-histogenetic, organization. A spatial and temporal organization of the proliferation activity is crucial in this last process. Four steps have been proposed to occur during the IsO appearance: positioning, induction, maintenance and morpho- histogenesis of the mid-hindbrain boundary and adjacent territories. This organized process depends on the operation of a cascade of molecular events that has been the subject of several excellent reviews (Ristoratore et al., 1999; Joyner et al., 2000; Rhinn and Brand, 2001; Wurst and Bally-Cuif, 2001; Hidalgo-Sánchez et al., 2005). A fifth step corresponds to the patterning of the OT alar plate and the final events correspond to the development of a terminally differentiated structural and functional organization. The end point is the establishment of the appropriate OT connectome.

The ordered occurrence of these steps depends on the operation of a multileveled network of protein interactions composed of several secreted proteins (Fgf8, Wnt1/3a/10b), their corresponding receptors and downstream effectors and several TFs such as Otx2, Meis, Pax2/3/5/7/8, En1, En2, and Lmx1b (Joyner, 1996; Echevarría et al., 2003; Mason, 2007). Several of these proteins form robust positive feedback loops while some antagonists operate as negative feedback loops on Fgf8 (Sef, Spry1, Spry2, and Mkp3) and localize the IsO activity at the M-H boundary. These proteins operate in a defined sequence whose final step is the OT localization and determination (Wurst and Bally-Cuif, 2001; Takashima et al., 2008; Agoston et al., 2012).

##### First step: Positioning of the M-H boundary

**FIGURE 8:**
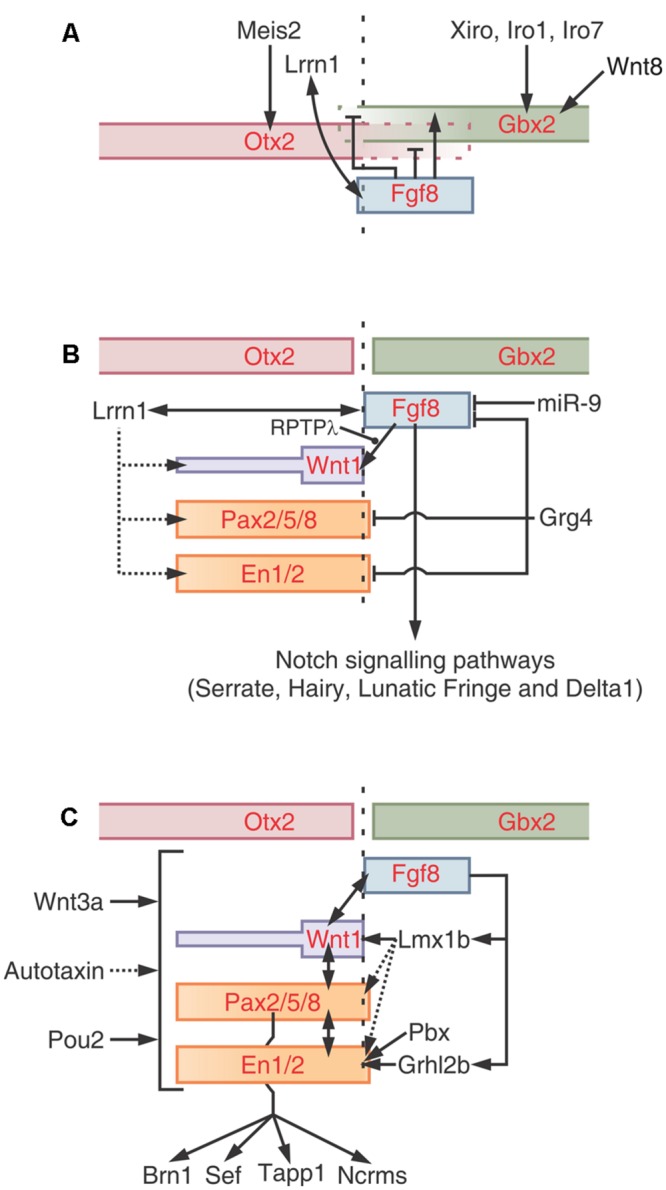
**Positioning, induction and maintenance of the isthmic organizer (IsO). (A)** Positioning of the M-H boundary. The zone of overlapping of Otx and Gbx defines the M-H boundary. After the neural plate “caudalization” by Wnt8, Otx2 is expressed in the future midbrain and Gbx1/2 in the future hindbrain. Meis2 regulates Otx2 expression in the midbrain whereas members of the Iroquies family regulate the expression of Gbx1/2 in the hindbrain. Fgf8 refines the Otx/Gbx interface by inhibiting Gbx1/2 expression in the midbrain and Otx2 expression in the hindbrain, respectively. Lrrn1 is involved in creating a sharp demarcation by creating a non-mixing zone between cells of future midbrain and hindbrain territories. **(B)** Induction of the IsO epigenetic program. The expression of the “classical” members of the IsO program is regulated at multiple levels. Fgf8 plays a central role inducing the expression of Wnt1 and other factors. The receptor protein tyrosine phosphatase λ (RPTPλ) regulate the Fgf8-induced activation of Wnt1. The microRNA miR-9 negatively regulates the Fgf8 pathways while proteins of the Iroquois family promote Fgf8 expression. Fgf8 and Lrrn1 are reciprocally regulated during the IsO cascade activation. Probably, Lrrn1 also interacts with En, Pax or Wnt proteins. The model shows that Grg4 also regulates the activity of several core members. The Notch signaling pathways (Serrate, Hairy, Lunatic Fringe, and Delta1) is expressed downstream Fgf8. **(C)** Model of the maintenance phase. Apart from the core IsO program (Fgf8, Wnt1, Pax2/5/8, and En1/2) several other factors are postulated to regulate the cascade. Some factors, such as Wnt3a, Autotaxin and Pou2, seem to globally regulate the cascade. Other factors, such as Lmx1b, Pbx, and grhl2b exert their effects on specific genes within the cascade. Two interactive loops that lie downstream Fgf8, mediated by Lmx1b and Grhl2b, also operate to maintain the expression of Wnt1 and En1/2. It is likely that Lmx1 also influence the expression of Pax and En transcription factors. Some putative maintenance factors, such as Brn1, Sef, Tapp1 and Ncrms, lie downstream of Pax2. Redrawn and modified from Dworkin and Jane (2013).

The first step depends on the spatially organized expression of the TFs Otx2 and Gbx2 along the cph→cd axis. In fact, the M-H boundary is specified at a small zone where the expression domains of Otx2 and Gbx1/2 overlap (**Figure 8A**). This phenomenon is part of the neural plate “caudalization”; a process mediated by Wnt8 signaling that probably proceeds from non-axial mesendoderm underlying the neural plate. It is likely that the TF protein Meis2 plays a role at this moment by de-repressing the Otx2 transcriptional activity and contributing to its activation at the M-H boundary. It was proposed that Fgf8 also plays a role in demarcating the Otx2/Gbx2 interface since (a) its expression domain is specifically restricted to the area of Otx2/Gbx2 interface overlapping and (b) it forms a feedback loop that spatially regulates the expression of both Otx2 and Gbx2 in the regions surrounding the future M-H boundary. In fact, Fgf8 activates Gbx2 and represses Otx2 at the isthmic region and suppresses Otx2 expression in the hindbrain.

The M-H boundary demarcates the limit between the midbrain and hindbrain primordia and, once established, the cells of these territories do not intermix anymore. The transmembrane boundary-demarcating protein Lrrn1 interacts with Fgf8 at the M-H boundary and this loop may contribute to its demarcation. It was proposed that Lrrn1 produces a process of differential cell adhesion at the boundary since Lrrn1 is exclusively expressed in the midbrain side of the boundary and not in the anterior hindbrain region. It is considered that, apart from all these proteins, other instructional cues are required to initiate the IsO program at the zone where the Otx2+ and Gbx2+ territories overlap. As an example, proteins of the Iroquois family are co-expressed with both territories during the M-H boundary positioning.

##### Second step: Induction of the IsO epigenetic program at the M-H boundary

This step is characterized by the activation of the Fgf8 and Wnt1 signaling pathways and the expression of a set of TFs such as Pax2, 5, 8, En1, 2 and others, at the zone of the Otx2/Gbx1/2 overlapping. These proteins are “classical” or core components of the IsO epigenetic program and several novel, “non-classical,” molecules modulate the expression of the core-IsO components (an excellent review is found in Dworkin and Jane, 2013).

A sequence of events has been postulated to explain how the information provided by the Otx2/ Gbx1/2 interface activates the downstream IsO program. One of the first steps in inducing the IsO program seems to be a Meis2-mediated change in the activity of the Otx2. It is known that Otx2 is inhibited by Grg4 which negatively regulates the expression of the IsO program. Meis2 interacts with Otx2 and competes with the Groucho co-repressor protein Grg4 for binding to Otx2, thereby releasing Otx2 from Grg4 mediated repression (Sugiyama et al., 2000).

Fgf8 is considered a “top” component of this step due to its ability to induce the IsO program and to promote the formation of both tectal and cerebellar tissues when implanted in competent neural regions (Crossley et al., 1996). Given its centrality, the Fgf8 signaling activity is finely regulated to ensure an appropriate inductive potential on the tissues surrounding the IsO. In fact, Fgf8 does not operate individually but within a network of protein interactions that includes the expression of the classical components (Pax, En, and Wnt families) and several additional regulatory factors. A model of the networks of interactions involved in the regulation of this step is shown in **Figure 8B**. It must be noted that the network includes several positive and negative loops.

Several signaling pathways, such as the Notch pathways, are activated upstream of Fgf8. It is not clear whether these pathways regulate the induction or the maintenance of the IsO program.

##### Third step: Maintenance of the IsO epigenetic program

The maintenance phase depends on different kind of interactions between classical and non-classical components: (a) reciprocal interactions between the classical members (Otx, Gbx, Fgf8, Wnt, Pax, and En), (b) several loops operating upstream Fgf8 and (c) several, non-classical, maintenance factors that globally regulate the IsO program acting on classical members (**Figure 8C**). Alterations in the expression or activity of any of the core components lead to alterations in the expression of the other factors followed by disruption of the IsO and subsequent patterning anomalies (Ristoratore et al., 1999; Rhinn and Brand, 2001; Dworkin and Jane, 2013).

Maintenance factors are defined as proteins that do not participate in the initial induction of the IsO program but their alterations produce a rapid decrease in the expression of these proteins. It is thought that they generate a permissive context for the optimal expression of the IsO program.

Examples of maintenance interactions are shown in **Figure 8C**. The protein Grhl2b acts downstream of Fgf8 and regulate the transcription of En2a. In brief, Fgf8 phosphorilates Erk which activates the Grhl2b, and this protein, in turn, activates En2a expression. In Grhl2b morphants embryos, the expression of the core components, except for En2a, are not affected during the induction phase. Later, during the maintenance phase, the expression of the core components drastically decreases indicating that the initial alteration in the En2a later affects the expression of the remaining components. Another example of a maintaining loop is mediated by Lmx1b which is necessary for the sustained expression of Wnt1 after its activation by Fgf8.

Wnt3a also plays a maintaining function since its deficiency does not alter the induction of the IsO cascade components but after the induction they rapidly disappear. The Fgf family, namely the Fgf8, continues playing a central role in maintaining the M-H boundary and the IsO.

**Figure 8C** shows a model of the IsO program maintenance (Dworkin and Jane, 2013). The model includes several other factors that are considered to play significant roles in maintaining the expression of one or more members of the core-IsO cascade. The model also incorporates some factors, such as Brn1, Sef, Tapp1 and Ncrms, that lie downstream of the core-IsO cascade, in this case, downstream Pax2. It is not clear whether these factors operate as maintenance factors or operate as nexus with the following (M-H boundary morphogenesis) step.

##### Forth step: The midbrain localization – OT localization and determination

The OT localization and determination is a “local” process included within a more general process: the patterning of the cephalic area (encephalon) of the neural plate. The CNS pattern is installed gradually from the most general to the most particular aspects. First, the main regions are established and afterward the sub-regions and their structural details are defined. This organized process is controlled by several organizers with different hierarchy.

**FIGURE 9:**
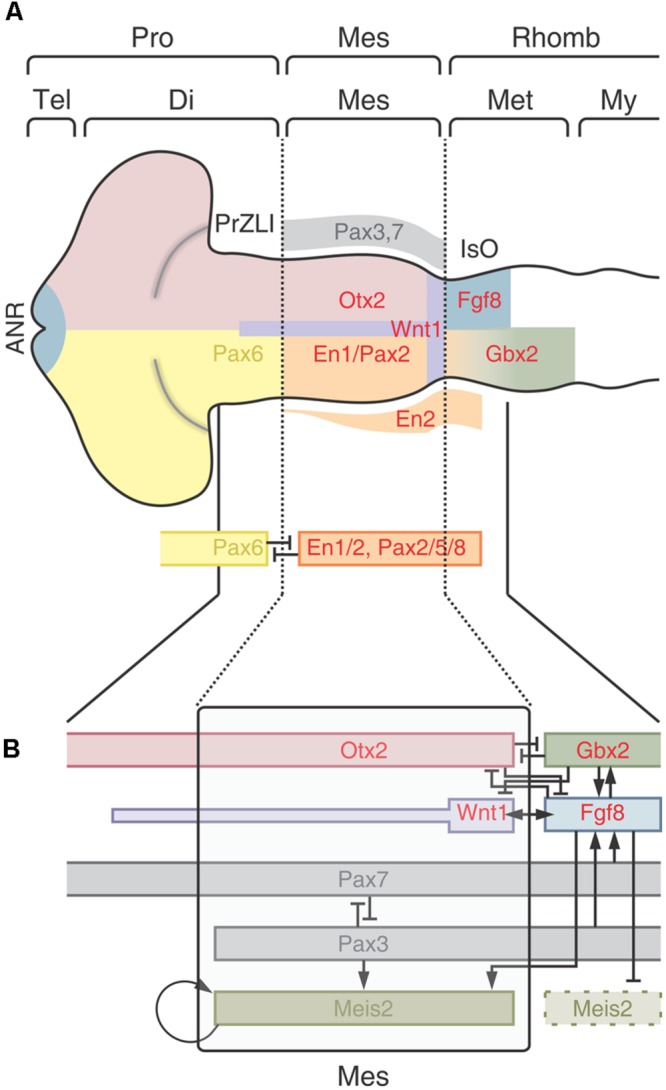
**The optic tectum localization and determination. (A)** Patterning of the encephalon. The regionalization depends on the spatially organized combinatorial expression of TFs that determine the primary CNS regions and sub-regions. Dotted lines represent the cephalic and caudal limits of the OT primordium. Pro, Prosencephalon; Tel, telencephalon; Di, diencephalon; Mes, mesencephalon; Rhomb, rhombencephalon; Met, metencephalon; My, myelencephalin; ANR, anterior neural ridge; PrZLI, presumtive zona limitans intrathalamica; IsO, isthmic organizer. **(B)** The model proposes that the TALE (three amino acid loop extension) homeodomain protein Meis2 plays role in assigning identity to the OT primordium. A network of protein interactions restricts the expression of Meis2 to the dorsal midbrain and the physical interactions between Meis2 and three TFs -Otx2, Pax3, and Pax7- specify and localize the tectal identity. Meis2 acts downstream of Pax3/7 and depends on a balanced activity of both factors which, reciprocally inhibit each other’s expression. Both Pax3 and Pax7 also induce Fgf8 expression. Although, Fgf8 is inhibited by Otx2 in the tectal primordium domain, the moderate Fgf8 signaling activity in the OT primordium is enough to stimulate Meis2 expression. Meis2 autoregulates its own expression and thereby stabilizes the OT determination. Redrawn and modified from Agoston et al. (2012) and Nakamura (2013).

The patterning implies the installation of different regional identities and, despite of its complexity, most scientists agree that the entire process is a remarkably well-conserved evolutionary process (Lowery and Sive, 2009; Hirth, 2010). **Figure 9A** shows a model of the spatially organized combinatorial expression of TFs that determine the primary CNS regions and sub-regions in the cephalic neural plate. The position of the main organizers is also shown.

One of the earliest events in the neural plate patterning is the appearance of a cephalic Otx2+ and a caudal Gbx2+ domains. Otx2 expression begins during gastrulation, rostral to the Hensen’s node (Simeone et al., 1992; Wassarman et al., 1997; Nakamura, 2013) while Gbx2 is expressed posterior to the node (Shamim and Mason, 1998). During neurulation the Otx2 expression domain characterized the prosencephalon and the mesencephalon and Gbx2 is expressed in the metencephalic region (Dworkin and Jane, 2013). Due to its relevance in the positioning, induction and maintenance of the IsO, these TFs are centrally involved in the OT localization and determination.

**Figure 9A** shows, schematically, that the cephalic limit of the midbrain (di-mesencephalic boundary) is defined by the reciprocal repressive interactions between Pax6 and En1/Pax2. Pax6 expression is restricted to the prosencephalon and En1 and Pax2 are expressed in the midbrain and anterior hindbrain (Araki and Nakamura, 1999; Schwarz et al., 1999; Matsunaga et al., 2000). The caudal limit of the midbrain is defined by the M-H boundary as it was described in preceding sections.

Once established at the M-H boundary, the IsO organizes the patterning of the adjacents OT (dorsal midbrain) and cerebellum (dorsal anterior hindbrain or metencephalon). **Figure 9B** shows that the synchronized and overlapped expressions of Fgf8, Meis2, Otx2, Pax3, and Pax7 compose a network of protein interactions that localize and determine the OT in the dorsal midbrain (Matsunaga et al., 2001; Vennemann et al., 2008; Agoston and Schulte, 2009). Although the precise spatio-temporal expression of these proteins may vary between vertebrate species (Rhinn and Brand, 2001), and different gene orthologs may perform the same function (Dworkin and Jane, 2013), studies in several species converge to show that a general model, with similar patterns of protein interactions can be proposed to operate at the IsO of most vertebrate species [teleosts (Moens and Prince, 2002; Jászai et al., 2003), amphibians (Glavic et al., 2002; Bandín et al., 2013), birds (Martinez et al., 1991; Itasaki and Nakamura, 1992); and mammals (Liu and Joyner, 2001)].

#### Establishment of the Dorsal-Ventral Patterning

The midbrain dorsal-ventral patterning is regulated by the combined organizing activity of IsO and the floor and roof plates. In the chick embryo OT, this polarity is installed from HH12 onward. By HH16 the OT has acquired a fixed dorsal-ventral identity and the region-specific gene activity become independent from the organizers (Li et al., 2005). This process takes place at a stage when the OT wall is entirely composed of pluripotent NScs. Thus, the dor→ven patterning generates a spatially organized distribution of NScs subpopulations with different region-specific identity. Apart from this dor→ven patterning, space-dependent differences in Fgf signaling activity along the OT primordium cph-cd axis install regional specificities in the rostro-caudal axis. As an example, in the mouse, specification of IC progenitors requires a higher level of Fgf signaling than specification of SC progenitors (Basson et al., 2008). Following patterning, several signaling pathways and their target TFs control the rate and the spatial organization of the proliferative activity. Some of these pathways and TFs are analyzed in following paragraphs.

### Temporal Regulation and Spatial Organization of Neural Stem Cell Proliferation

In the simplest scenario, the proliferative phase of the neuroepithelium is composed of at least three, partially overlapped, phases: (**a**) a first phase of expansive proliferation; NScs divide symmetrically, the number of NScs increases exponentially and the neuroepithelium expands tangentially; (**b**) a phase of asymmetric neuronogenic proliferation; this phase is subdivided in subphases and (**c**) a last phase of gliogenic proliferation.

It is clear that the proliferative activity begins and is spatially organized around the IsO (Rapacioli et al., 2012b). This temporal-spatial organization requires modulating the proliferation rate, time of cell cycle exit, onset of determination/differentiation etc. These parameters are regulated as a function of (**a**) the time and of (**b**) the position along cph-cd and med-lat axes. Several sets of proteins (growth factors, receptors, TFs and other signaling proteins) participate in this regulation (Ahmed et al., 2009). Naturally, growth factors act upstream their receptors, their downstream effectors and their target TFs, and also upstream of the cell cycle controlling proteins (cyclins, kynases etc.). Several signaling pathways and their targets TFs have been identified and exhaustively studied in the developing OT in several species. **Table 4** summarizes these data.

**Table 4:** Summary of signaling pathways and transcription factors involved in the temporal and spatial regulation of cell proliferation.

Signaling pathways		Transcription factors	
Fgf8	Specification Patterning Promotion of dorsal midbrain cell survival	Id Hes NICD	Maintaining the proliferative status Repression of neuronal differentiation
Fgf15/19 Fgf21 Fgf23	Inhibition of cell proliferation Promotion of neuronal determination/differentiation	Proneural TFs	Inhibition of cell proliferation Promotion of neuronal determination
Wnt	Specification Patterning Maintaining of the proliferative status Triggering of neuronal differentiation	NeuroD	Promotion of neuronal differentiation
Shh	Patterning (basal plate) Promotion of cell survival and proliferation (alar plate)	Pax7	Specification Patterning Promotion of cell survival/differentiation
Notch	Maintaining the proliferative status Repression of neuronal differentiation		

#### Signaling Pathways

##### Fgf family and Fgf signaling pathways

The fibroblast growth factor (Fgf) family is composed of many members. Most of them stimulate proliferation, maintain the proliferative status of neuroprogenitors (Mason, 2007; Partanen, 2007; Lahti et al., 2011) and promote cell survival. Some of them, however, inhibit proliferation, driving differentiation (Borello et al., 2008).

The Fgf8 secreted by the IsO participates in morphogenesis of the midbrain and the cerebellum (Martínez, 2001; Mason, 2007). These primordia require different levels of Fgf signaling for their specification and patterning (Sato et al., 2001; Liu et al., 2003) and the establishment of different levels is regulated by the Sprouty proteins (Hacohen et al., 1998; Casci et al., 1999). In turn, Sprouty expression is induced by signaling through Fgf receptors and other receptor tyrosine kinases (Mason et al., 2006; Basson et al., 2008). At the beginning of the OT development, Fgf8 is widely distributed along the Cph-Cd axis. Later, its expression is restricted to the rostral most metencephalic region (Ishikawa et al., 2008). Space-dependent differences in the level of Fgf8 signaling activity along the cph-cd axis mediate the differential determination of IC and SC in mammals (Basson et al., 2008).

Fgf signaling also regulates dorsal midbrain’s cell survival. There is a minimum level of Fgf signaling below which dorsal midbrain’s cells die. However, the Fgf secreted at the IsO is enough to sustain cell survival along the entire dorsal midbrain cph-cd axis. As an example, the Sprouty2 gain-of-function mutant mouse undergoes a moderate reduction in Fgf signaling and only the cells near to the IsO survive (Basson et al., 2008).

Some members of the Fgf family (Fgf15/19, Fgf21, and Fgf23) belong to an atypical subfamily that inhibits proliferation and promote neuronal determination/differentiation (Borello et al., 2008; Dyer et al., 2014). They display low-affinity heparin-binding sites and require Klotho/β-Klotho transmembrane proteins for signaling via Fgf receptors (Itoh and Ornitz, 2008; Jones, 2008). Their transcription is regulated by members of the nuclear receptor class of ligand activated TFs. It is also regulated by the Shh signaling pathway, as described below (Saitsu et al., 2005).

Fgf15 possesses a well-defined area of expression in the dorsal midbrain; it inhibits the expression of several TFs that maintain the proliferative status (Id1, Id3, and Hes5 HLH TFs) and promotes the expression of proneural TFs [Ascl1 (Mash1), Neurog1 (Ngn1) and Neurog2 (Ngn2)] and neuronal differentiation TFs (NeuroD). In this way Fgf15 regulates the transition from the proliferative status to neural commitment and initiates neuronal differentiation. In the mutant mouse embryo (Fgf15-/-) dorsal midbrain neuroprogenitors fail to exit the cell cycle, generate an excessive amount of neurons and produce an overgrowth of the alar plate (McWhirter et al., 1997; Bertrand et al., 2002; Ishibashi and McMahon, 2002; Gimeno et al., 2003).

##### Wnt family and Wnt signaling pathways

Several members of the Wnt family are expressed in the dorsal midbrain. During the early stages, the Wnt/β-catenin pathway displays a broad expression area in the SvZ of the dorsal midbrain (Kalani et al., 2008). Later, its expression is reduced to the caudalmost region and the wnt1-positive cells, rostrally to the isthmic constriction, define the midbrain caudal limit (Ishikawa et al., 2008).

Members of the Wnt family exhibit versatile roles during neuronogenesis. They promote neurogenesis maintaining the proliferative status of neuroprogenitors and repressing their differentiation or, by contrast, promote neuronal fates specification in a time- and context-dependent manner (Megason and McMahon, 2002; Hirabayashi et al., 2004; Kuwabara et al., 2009; Wexler et al., 2009). In fact, depending on the developmental stage the Wnt/β-catenin pathway switches its role into triggering neuronal differentiation (Hirabayashi et al., 2004). It has been proposed that β-catenin, associated to Fgf2, inhibits neuronal differentiation but acting alone stimulates neuronal differentiation (Israsena et al., 2004). β-catenin and its downstream effectors (TCF/LEF) are involved in regulating the balance between progenitor expansion and differentiation (Zechner et al., 2003; Shimizu et al., 2008).

##### Shh signaling pathways

Shh also exhibits a versatile role, acting as a morphogen in the ventral basal plate (Li et al., 2005; Bayly et al., 2007) and as a mitogen in the midbrain dorsal alar plate (Agarwala et al., 2001; Feijóo et al., 2011; Rapacioli et al., 2012a; Martínez et al., 2013). Shh exhibits several developmental roles in the midbrain and metencephalon (r1) primordia and their derivatives (OT and cerebellum respectively; Dahmane and Ruiz i Altaba, 1999). During the early somitic period Shh stimulate Cyclin D1 expression in the ventral and dorsal midbrain and regulates cell proliferation and survival in the dorsal midbrain (Ishibashi and McMahon, 2002).

In the zebra fish OT, pharmacological loss-of- and gain-of-function of Hedgehog (Hh) signaling significantly reduces and increases cell proliferation respectively. These kinds of experiments, performed at different developmental stages, allow identifying a critical period of neuroprogenitors sensitivity to Hh signaling (Feijóo et al., 2011). Abolition of Hh signaling in mutant zebrafish reduces neuroprogenitors proliferation (Barresi et al., 2000; Varga et al., 2001; Karlstrom et al., 2003; Feijóo et al., 2011). In zebrafish, Gli1 is the major activator of Hh target genes while Gli2, similarly to Gli3, plays both activator and repressor roles in different regions of the embryo (Karlstrom et al., 2003; Tyurina et al., 2005).

Due to its effects on cell proliferation and survival, Shh signaling also affects midbrain growth and morphogenesis. In fact, during the late stages of the mouse embryo, Shh has an influence on the tectal midbrain growth (Dahmane et al., 2001; Palma and Ruiz i Altaba, 2004). In mice bearing a Shh mutation the spinal cord and brain, including the mes/r1 region, are severely reduced in size (Chiang et al., 1996; Fedtsova and Turner, 2001). Consistently, in the chick embryo, a transient decrease in Shh signaling reduces cell proliferation, increases cell death and produces a reduction in the midbrain size (Britto et al., 2002; Rapacioli et al., 2012a).

Analyses performed in Shh null mutant mice revealed that Shh is essential for the growth of both dorsal and ventral diencephalon and anterior midbrain regions (Britto et al., 2002; Ishibashi and McMahon, 2002). In this mutant, the ventral and dorsal diencephalon and the anterior midbrain have reduced proliferation and increased apoptosis (Ishibashi and McMahon, 2002).

Shh signaling regulates cell proliferation/apoptosis in the dorsal mes/diencephalon by influencing the expression of Bmp4 and Tcf4 (a component of the Wnt signaling pathway; Ishibashi and McMahon, 2002). Shh also influences Fgf15 activity whose expression in the mouse midbrain directly depends on the Shh/Gli signaling. In fact, the 3.6-kb Fgf15 enhancer/promoter is a direct target of Gli2 protein (Saitsu et al., 2005).

In the mouse embryo, gain-of-function experiments of Shh signaling produce an increase in cell proliferation and excessive growth in the dorsal midbrain and metencephalon. Similarly, the local application of Shh in the chick embryo midbrain produces an increase in cell proliferation (Agarwala et al., 2001). *In ovo* Hh loss-of-function treatments using the alkaloid cyclopamine results in reduced OT expansion while gain-of-function with the co-receptor smoothened agonist purmorphamine produces larger OT. Besides, Shh or Gli1 electroporation increase proliferation in the dorsal regions and abolishe or interfere with the intertectal sulcus formation respectively (Rapacioli et al., 2012a).

##### Notch and BMP pathways

The Notch signaling is also implicated in regulating the balance between the expansive proliferation of NScs and neuronal determination and differentiation (Gridley, 1997; Kageyama et al., 2005). The Notch signaling activity, on one hand, stimulates Hes1 and Hes5 expression, promotes proliferation and maintains proliferating cells in an undifferentiated state and, on the other hand, represses neuronal differentiation (Ohtsuka et al., 2001; Sakamoto et al., 2003; Hatakeyama et al., 2004). In mammals, Musashi-1 increases the Notch signaling activity through the translational repression of its target mRNA, mNumb, thereby contributing to the maintenance of NScs and progenitors (Okano et al., 2005).

Finally, bone morphogenetic protein (Bmp)/transforming growth factor β (Tgfβ) family members are also implicated in regulating cell proliferation in the developing OT since they have been reported to promote cell cycle exit of neural progenitors (Siegenthaler and Miller, 2005; Roussa et al., 2006).

#### Transcription Factors

Three sets of TFs are centrally involved in regulating (a) the duration of the cell proliferative status, (b) the transition from the symmetric to asymmetric proliferation and the relative duration of each phase, (c) the time of cell cycle exit and (d) the onset of neuronal commitment and differentiation (Guillemot, 2007):

(**a**) A group of TFs, such as Id, Hes, NICD (Notch intracytoplasmatic domain) and others, maintain the proliferative activity of NScs and neuroprogenitors. These TFs directly inhibit the expression of proneural TFs maintaining an uncommitted and undifferented status of proliferating cells. These TFs are intensely expressed in the generation zone of the OT neuroepithelium during the early proliferative phase (Rapacioli et al., 2011).

(**b**) A second group of TFs, generically designated as “proneural TFs,” such as Ascl1 (Mash1) and Neurogenins (Neurog, Ngn), promotes the cessation of the proliferative activity and the cell cycle exit of neuroprogenitors. These TFs also promote neuronal determination and the activation of Notch signaling in adjacent progenitors.

(**c**) Another group of TFs initiates the program of neuronal differentiation. The expression of these TFs is induced by the proneural TFs. Once committed, the post-mitotic neurons exit the cell cycle and TFs, such as Neurod1 (NeuroD), initiate the neuronal differentiation. At the beginning of neuronogenesis, NeuroD is highly expressed in post-mitotic neurons distributed in the sVz and premigratory zones of the OT neuroepithelium (Rapacioli et al., 2011). Some TFs are specific for some neuronal layers. As an example, a comparative study of the SC development in Pax7 mutant and wild type mice demonstrates that Pax7 is required to maintain a subpopulation of dorsal midbrain neurons. Besides, Pax7 partially regulates the spatiotemporal expression of Pax3 and the expression of both coincides spatiotemporally with neuronal differentiation and tissue maturation. The Pax3 expression is perturbed in the CNS of Pax7 mutant mice embryo. A comparative analysis of embryonic Pax3 and Pax7 expression profiles indicate that initially the Pax3 expression area overlaps extensively with that of Pax7; later, as development progresses, their expression domains diverge (Thompson et al., 2004, 2008).

#### Cell Cycle Control Proteins

Many of the groups of molecules described in precedent paragraphs are upstream of non-neuron-specific cell cycle controlling proteins such as the cyclin-dependent kinase inhibitor (cdki) Cdkn1b (p27Kip1) and the retinoblastoma (Rb) proteins Rb1, Rbl1 (p107), and Rbl2 (p130). These proteins are also involved in regulating the cell cycle exit and differentiation (Galderisi et al., 2003; Nguyen et al., 2006).

## Hypothesis About the Optic Tectum Evolution

Corticogenesis depends, amongst other phenomena, on the appropriate temporal-spatial organization of several developmental events: (**a**) the duration of the proliferative phase of the OT neuroepithelium, (**b**) the relative duration of symmetric (expansive) versus asymmetric (neuronogenic) subphases, (**c**) the dynamics and (**d**) the spatial organization of each kind of cell division, (**e**) the time of determination and cell cycle exit and (**f**) the time of onset of the post-mitotic neuronal migration and (**g**) the time of onset of neuronal function.

Of all these events, the first five directly refer to the regulation of cell proliferation. Changes in any of them could significantly influence the cortical extension, its morphology, its architecture and finally its wiring (arriving of afferent, formation of local circuits, and efferent projections) and normal function. In fact, there is a significant amount of cortical congenital disorders caused by alterations in cell proliferation.

Apart from some basic similarities, the OT cortex development also exhibits several significant species-dependent differences. Some of them seem to arise from differences in the mode the NSc proliferation is regulated during development.

In spite of the fact that most of the proteins involved in the cell cycle regulation, i.e., the proteins comprising the cell cycle control system- exhibit a high degree of conservation along evolution, the network of proteins interactions that defines the cell cycle control systems exhibit significant species-dependent functional peculiarities. It is plausible thinking that these differences resulted from evolutionary changes in the mode of operation of proteins networks involved in regulation of NScs proliferation, i.e., the protein network comprising the cell cycle control system (Rakic, 1995; Kriegstein et al., 2006; Caviness et al., 2009).

**FIGURE 10:**
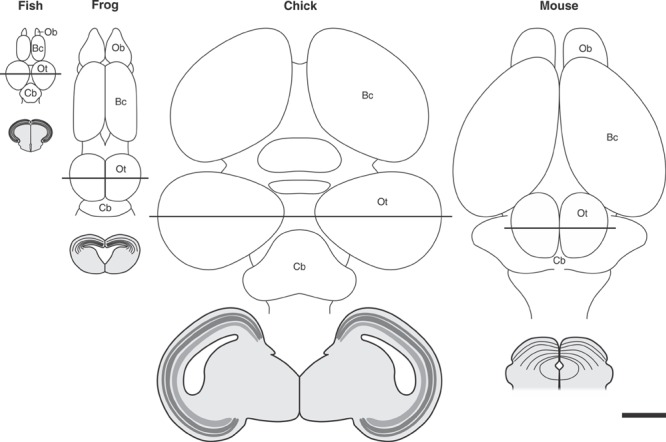
**Tangential expansion the OT cortex from teleost to birds.** The upper panel shows schematic representations of dorsal aspect of the encephalon of different vertebrate species. Transverse sections of the midbrain (indicated by the continuous straight lines) are illustrated below each figure. Significant differences in tangential (planar) expansion and thickness can be observed from teleosts to birds. The reduction observed between birds and mouse could be due to the transfer of many OT functions to the brain cortex during evolution. Ob, Olfactory bulb; Bc, brain cortex; OT, optic tectum; Cb, cerebellum. All images were drawn at the same magnification: Bar = 2 mm. Redrawn and modified from Raymond and Easter (1983), Lowe (1986), Luksch (2003), and Isa and Hall (2009).

Several generic models of corticogenesis and cortical evolution propose that the tangential expansion the OT cortex has underwent during evolution could have involved a lengthening in the symmetric (expansive) phase of NScs proliferation. This evolutionary change could have led to an increase in the number of symmetric cell cycles by maintaining a long-lasting population of NScs. This phenomenon could, in turn, result in an increase in the number of neuronal cohorts (radial columnar units) added to the tangential (cph-cd/lat-med) plane and, as a direct consequence, a tangential expansion of the OT primordium during evolution (**Figure 10**).

This speculation rests on the fact that, during ontogeny, in any species analyzed, the degree of OT tangential expansion depends on how long the OT sustains an actively proliferating NSc population and on the number of cycles performed during that period. In fact, the tangential expansion underwent by the OT during embryonic development, and also during the adult growth in teleost fishes, depends on the maintenance of a proliferative zone populated by NScs and neuroprogenitors [mpz (in teleosts), the caudal-medial zone (in amphibians) and the ZHMD (in the chick embryo)]. In all cases it holds that the longer the symmetric sub phase of proliferation and the higher the NSc proliferation rate, the greater the OT tangential expansion.

With regards to the organization along the OT radial axis, it is usually considered that both the thickness and the density of cells of the whole cortex and of each cortical layer depend on three main parameters: (**a**) the proliferation rate, (**b**) the length of the neuronogenic period and (**c**) the temporal-spatial pattern of neuronal migration.

After entering the neuronogenic phase, the number of neurons produced locally, at each “point” of the ventricular zone, depends on the number of asymmetric division performed by each neuroprogenitor. Given the absence of significant tangential dispersion during the radial migration, the number of neurons of each radial cortical unit (cortex column) is strongly determined by the number of “local” neuronogenic cell cycles.

It must be noted that, apart from some basic similarities in the OT lamination (stratification) there are significant inter-specific differences in the OT organization and complexity along the radial axis.

It has been pointed out (Rakic, 1995) that the proteins involved in the cell cycle control appear to be remarkably conserved across species (Murray and Hunt, 1993; Wolpert, 1994) and that the inter-specific differences could depend rather on differences in the neuronogenic phase duration and proliferation rate during that period.

Changes in the regulation of radial cell migration must also be considered to explain the inter-specific differences in stratification. In fact, the relative occupancy (cell density) of each neuronal layer strongly depends on the pattern of post-mitotic neuronal migration. There are significant changes in complexity along the radial axis (lamination and neuronal density) from urodeles (two layered) to the birds (16 alternating layers; **Figure 1**). It has been pointed out that, from teleosts onward, the OT evolution has involved a tangential expansion -due to the addition of columns of cells- and a relative increase in thickness and complexity along the radial axis. It has also been postulated that teleosts fishes already achieved a basic cortical stratifications and that later, specific functional adaptations associated to the process of speciation and adaptation to different environments have introduced changes in the mechanisms regulating the OT cortex ontogeny. In this context the simplicity observed in *Pleurodeles* has been interpreted as a paedomorphic simplification. The simplification observed from birds to mammals was postulated to be the result of a specialization and transference of the visual functions to the brain visual cortex.

In considering generic strategies that could account for the cortices evolution Rakic (1995) postulated that “mutation of a regulatory gene(s) that controls the timing and ratio of symmetric and asymmetric modes of cell divisions in the proliferative zone, coupled with radial constraints in the distribution of migrating neurons, could create an expanded cortical plate with enhanced capacity for establishing new patterns of connectivity that are validated through natural selection.”

## Concluding Remarks

It is presumed that the modern neurosciences will accumulate enough information as to describe the human mind in terms of the function of interconnecting cortical and subcortical neuronal circuit networks. While this could be a long-range goal, a less ambitious objective could be the description of the behaviors of lower species in terms of defined patterns of activity of the above mentioned neuronal circuits.

A fashionable idea that has generated a great expectation in the fields of the psychology, cognitive neurology, sociology and so on, is that the overall human behavior, including the consciousness with its emotional and moral components will be explained, in the not too distant future, in terms of the networking of the highest cortical areas and their corresponding subcortical centers.

This “neural-based notion of behavior” is subsumed in the modern notion of connectome. According to this view each kind of specific behavior is determined by (a) the existence of specific patterns of connections between different regions (cortical areas and subcortical nuclei) and (b) characterized by defined patterns of global neural activity. The connectome can be envisioned as the “wiring of the CNS” but the “wires” are highly dynamics and plastic elements whose structure and functions are continuously modified by the network activity itself. Similarly to any computer system, the quality and efficiency of the information processing critically depends on the number of inter-connected elements, their positions, their distances between them, the speed of the flow of information between elements and the enrichment of connections between these centers.

The relevance of the information and the experimental approaches presented in this review derives from the fact that they help to more precisely understand the initial spatial organizations of the elements (different neuronal subpopulations) that after subsequent phases of differentiation, i.e., dendrogenesis, axonogenesis, synaptogenesis, etc., will generate the specificities of each local subdomain of a global connectome.

It is advisable that future research in the field of developmental neurobiology should be aimed at elucidating the mechanisms by which the CNS executes a finely regulated process of neuronogenesis and neuronal localization along the three spacial axes of the system. This is a process that interactively involves different kind of cell proliferation and several kinds of post-mitotic neuronal migration. The understanding of how the primary spatial organization and distribution of the different neuronal populations composing the encephalic vesicles is established is the first step for the generation of a simple model for subsequent connectomics analyses. In fact this information is necessary to subsequently understand how neuritogenesis and synaptogenesis are spatially organized to generate the raw pattern of connectivities that, after an experience-dependent refinement, remains as the cortical and subcortical subdomains of the global connectome.

## Author Contributions

MR, VP, and VF wrote the manuscript.

## Conflict of Interest Statement

The authors declare that the research was conducted in the absence of any commercial or financial relationships that could be construed as a potential conflict of interest.
