# Corrigendum: Morphogenetic and Histogenetic Roles of the Temporal-Spatial Organization of Cell Proliferation in the Vertebrate Corticogenesis as Revealed by Inter-specific Analyses of the Optic Tectum Cortex Development

**DOI:** 10.3389/fncel.2016.00112

**Published:** 2016-05-09

**Authors:** Melina Rapacioli, Verónica Palma, Vladimir Flores

**Affiliations:** ^1^Interdisciplinary Group in Theoretical Biology, Department of Biostructural Sciences, Favaloro UniversityBuenos Aires, Argentina; ^2^Laboratory of Stem Cell and Developmental Biology, Faculty of Science, University of ChileSantiago, Chile; ^3^Laboratory of Developmental Neurobiology, School of Medicine, Institute of Cell Biology and Neurosciences “Prof. E. De Robertis” (UBA-CONICET), University of Buenos AiresBuenos Aires, Argentina

**Keywords:** cell proliferation, corticogenesis, morphogenesis, histogenesis, patterning

One of the affiliations of Vladimir Flores was omitted in the original paper.

The original paper affiliations were:

*Melina Rapacioli*^*1*^*, Verónica Palma*^*2*^ *and Vladimir Flores*^1*^

^*1*^*Interdisciplinary Group in Theoretical Biology, Department of Biostructural Sciences, Favaloro University, Buenos Aires, Argentina*
^*2*^*Laboratory of Stem Cell and Developmental Biology, Faculty of Science, University of Chile, Santiago, Chile*

The correct affiliations should be:

*Melina Rapacioli*^*1*^*, Verónica Palma*^*2*^ *and Vladimir Flores*^1, 3*^

^*1*^*Interdisciplinary Group in Theoretical Biology, Department of Biostructural Sciences, Favaloro University, Buenos Aires, Argentina*
^*2*^*Laboratory of Stem Cell and Developmental Biology, Faculty of Science, University of Chile, Santiago, Chile*
^*3*^*Laboratory of Developmental Neurobiology, Institute of Cell Biology and Neurosciences “Prof. E. De Robertis” (UBA-CONICET), School of Medicine, University of Buenos Aires, Buenos Aires, Argentina*

The correction does not affect the scientific validity of the results.

## Author contributions

All authors listed, have made substantial, direct and intellectual contribution to the work, and approved it for publication.

### Conflict of interest statement

The authors declare that the research was conducted in the absence of any commercial or financial relationships that could be construed as a potential conflict of interest

